# Experiences and Educational Needs of Hospital Staff Providing Care to Tracheostomy-Dependent Pediatric Patients

**DOI:** 10.3390/children12050552

**Published:** 2025-04-25

**Authors:** Kathryn L. Palumbo, Desirae Smith, Adrianne Frankel, Laine DiNoto, Taylor Wheaton, Kimberly Buholtz, Rita Dadiz

**Affiliations:** 1 Department of Pediatrics, University of Rochester Medical Center, Rochester, NY 14642, USA; desirae_smith@urmc.rochester.edu (D.S.); adrianne_frankel@urmc.rochester.edu (A.F.); rita_dadiz@urmc.rochester.edu (R.D.); 2 Department of Otolaryngology and Pediatrics, University of Rochester Medical Center, Rochester, NY 14642, USA; laine_dinoto@urmc.rochester.edu; 3 School of Nursing, University of Rochester, Rochester, NY 14642, USA; kimberly_buholtz@urmc.rochester.edu

**Keywords:** children, complex medical care, infants

## Abstract

Objective: To assess the experience and educational needs of hospital staff who care for pediatric patients with tracheostomies. Study Design: Staff were surveyed and participated in semi-structured, facilitated focus groups regarding their experiences caring for children with tracheostomies and their educational needs. Survey data were analyzed using descriptive statistics and Kruskal–Wallis nonparametric tests. Focus groups were transcribed verbatim and coded for thematic analysis. Results: Pediatric advanced practice providers, nurses, physicians, and respiratory therapists (152/353, 43%) completed the survey. Within the last year, 76% of staff had worked with a tracheostomy-dependent child. However, up to 59% of staff had not performed at least one tracheostomy skill (e.g., tracheostomy site assessment, tube change, etc.). Staff reported the least confidence in changing tracheostomy tubes and using home ventilators and rated these skills as most important for additional education. Forty-three staff members participated in 1 of 10 focus groups. Three themes were identified: building staff competencies in tracheostomy care, promoting the caregiver development of tracheostomy skills, and building caregiver preparedness for home life. Staff emphasized the need for participating in emergency simulations and developing their skills to better prepare caregivers for home life. They indicated a need to streamline the discharge process, gain knowledge of community resources, and develop a standardized team to provide discharge teaching. Conclusions: Hospital staff responsible for providing care to tracheostomy-dependent pediatric patients had limited opportunities to learn and maintain their skills. Survey and focus group findings can guide development of continuing education to optimize the care of tracheostomy-dependent children.

## 1. Introduction

Over 26,000 tracheostomies were performed in pediatric patients between 2003 and 2016 [1], and children with tracheostomies are increasing in prevalence, due in part from the increased survival of infants born with prematurity and congenital anomalies [2,3,4]. Tracheostomies provide life-saving airway patency and allow for mechanical ventilation in patients with chronic respiratory failure. They can reduce the risk of death, the occurrence of ventilator associated pneumonia (VAP), and the duration of mechanical ventilation [5]. They can also facilitate improved communication skills, oral hygiene, and the ability to swallow and drink/feed orally [5]. In these patients, tracheostomies also facilitate hospital discharge to home or a chronic care facility, leading to a decreased length of hospital stay for patients in the intensive care setting [5,6]. However, when compared with adults with tracheostomies, tracheostomy-dependent pediatric patients (TDPP) have higher rates of hospital readmission, morbidity, and mortality due to the complexity of their underlying medical condition and the smaller size of their airway anatomy [2,7,8,9]. Bleeding (3–7% of children), tracheostomy tube dislodgement (4%), and tracheostomy tube obstruction (7–16%) are among the most common early and late complications contributing to morbidity and mortality [10,11,12].

When tracheostomy-related emergencies occur in the hospital setting, frontline staff who comprise the patient’s primary team are usually the first to respond and stabilize the patient. However, because tracheostomies occur in less than 1% of children [12], staff confidence, knowledge, and experience working with these children vary widely [13,14,15,16]. Published clinical practice guidelines focus on the elements that should be included in caregiver education [17]. While a few countries have recommendations regarding the training of hospital staff in the care of adults with tracheostomies [18,19], there are no similar national or international guidelines for staff who work with TDPP. In addition, very limited studies have explored the experiences of hospital staff working with TDPP or surveyed them to identify their educational needs [20,21,22]. While some studies base curriculum design on a clinical consensus statement [23], recent studies that implement an educational intervention do not report the inclusion of a greater needs assessment to guide curriculum development [13,14,15,24,25,26,27]. Including frontline staff in curriculum design to understand their educational needs would help promote experiential learning grounded in adult learning theory and engage staff to identify relevant learning gaps, practice emergency response, and increase their support of families. We therefore conducted a mixed-methods needs assessment consisting of a survey and focus groups of pediatric staff who care for TDPP. We aimed to explore their experiences and educational needs that will help inform the development of an educational program to improve the knowledge, skills, and self-efficacy of hospital staff who work with TDPP.

## 2. Materials and Methods

### 2.1. Participants, Setting, and Approval

In 2021–2022, we conducted a mixed-methods study consisting of a survey and focus groups to ascertain staff confidence, clinical experiences, and educational needs to provide care to TDPP. Eligible staff included advanced practice providers, nurses, respiratory therapists, and attending and fellow physicians in the neonatal, pediatric, and pediatric cardiac intensive care units at the University of Rochester Medical Center, because they provide care to the majority of TDPP in the hospital. The University of Rochester institutional review board approved this study with a waiver of consent (study number 00005759). Study design followed the Standards for Reporting Qualitative Research [28]. 

### 2.2. Survey: Design, Distribution, and Data Analysis

The survey was designed, piloted, and iteratively revised by a multidisciplinary team of experts in neonatology, otolaryngology, and pediatric critical care following a systematic approach to survey design [29]. Questions focused on staff experience, confidence, and ongoing educational needs. Initial questions were developed by the multidisciplinary team based on their experiences caring for and working with staff who provided patient care to TDPP. These survey questions were piloted with a group of staff who did not participate in the study, and the questions were refined based on feedback. Utilizing REDCap for distribution [30], the final survey consisted of 13 questions using 6-point Likert scale ratings, multiple-choice options, and open-text responses (Supplementary Materials S1). Embedded links and QR codes were distributed at meetings and via email. Participation was anonymous and voluntary.

Demographic data are presented as descriptive statistics. Likert scale ratings are noted as medians with interquartile ranges (Q1, Q3). We utilized the Kruskal–Wallis nonparametric test to analyze Likert scale ratings based on staff years of experience working with TDPP. Data analyses were conducted with Statistics Kingdom [31]. Significance was accepted at *p* ≤ 0.05.

### 2.3. Focus Groups: Interview Guide, Staff Participation, and Data Analysis

We utilized convenience sampling to invite staff who completed the survey to participate in an in-person semi-structured focus group. Convenience sampling helped facilitate the scheduling of staff who had time constraints due to different and busy clinical schedules. The interview guide consisted of 17 open-ended questions to prompt staff reflection on their training, clinical experiences, and interactions with caregivers of TDPP (Supplementary Materials S2). We developed and iteratively revised the guide to ensure clarity and relevance of questions. Investigators (L.D., R.D., A.F., K.P., D.S., and T.W.) paired up to co-facilitate focus groups until thematic saturation was reached. Focus groups were audio recorded, transcribed verbatim using Rev Transcription (Rev.com, Austin, TX, USA), and checked for accuracy.

Utilizing the principles of grounded theory, two investigators coded each transcript independently, then discussed and refined codes [32,33]. They applied codes to subsequent transcripts and refined them further as needed. If they disagreed on a code, they involved a third investigator to reach agreement. All investigators participated in a series of immersion and crystallization cycles to draw contextual meaning, discuss themes and subthemes, and develop visual aids to conceptualize relationships [34,35]. To support the trustworthiness of data analyses, a fourth investigator reviewed the codes of 75% of transcripts and the audit trail of data analyses [32].

## 3. Results

### 3.1. Survey

**Figure 1:**
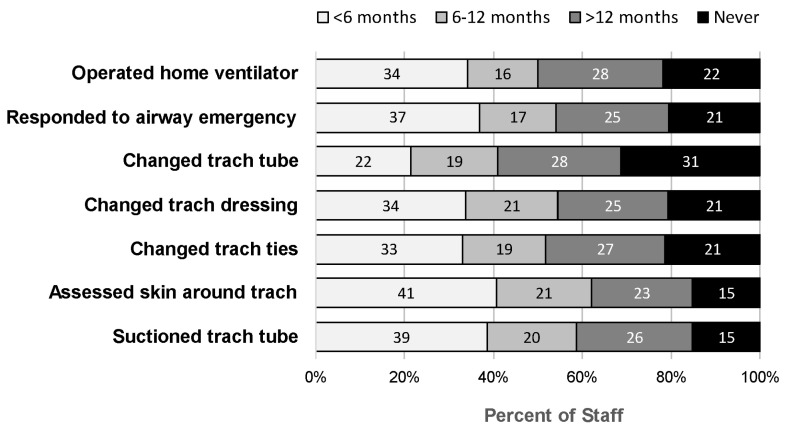
Staff Performance of Specific Tracheostomy Skills.

A total of 152/353 (43%) eligible staff completed the survey. Survey respondents had a range of tracheostomy experience (Table 1). Almost half practiced in their professional role for ≤5 years (66/152, 43%) and worked with TDPP for ≤5 years (74/152, 47%). Most (115/152, 76%) provided care to a TDPP within the last year (Table 1). The stacked bar chart (Figure 1) depicts the amount of time that has elapsed since staff performed different tracheostomy skills in the clinical setting. While 22–41% of staff had clinical experience within the last 6 months, 23–28% of staff had last performed these skills over a year ago. Another 15–31% of staff had never performed these skills.

While staff ranged in their experience, even staff with >10 years of experience reported that they were “slightly” or “moderately” confident in changing tracheostomy tubes [3 (3, 5)], using home ventilators [2 (2, 4)], and responding to alarms on a home ventilator [2 (2, 5)] (Table 2). Staff identified these skills as among the most important for receiving additional education (Table 3). In addition, they desired education on how to train caregivers (Table 3). In open comments, staff indicated a need to develop a standardized team to provide discharge teaching, streamline the discharge process, and gain knowledge of home and community resources.

To address gaps in tracheostomy knowledge and skills, staff (101/138, 73%) preferred interactive educational methods utilizing simulation and unit-based in-services over computer-based learning. When asked, “What concerns do you have or what changes would you like to make for the discharge process for tracheostomy dependent children”, respondents communicated in open comments the need for more simulation and “hands-on practice” for staff and caregivers.

### 3.2. Focus Groups

To better understand staff responses to survey questions, we conducted focus groups to allow for more in-depth discussion. We attained thematic saturation after conducting 10 focus groups that averaged 40 ± 10 min with 43 participants. Staff represented different professions and worked in various locations (Table 1). Data analyses revealed three themes (building staff competencies in tracheostomy care, promoting caregiver development of tracheostomy skills, and building caregiver preparedness for home life) and nine subthemes (italicized in sections below), representing major factors that contributed to staff experiences. Table 4 presents interview excerpts (Ex.) that illustrate these themes and subtheme.

### 3.3. Theme: Building Staff Competency in Tracheostomy Care

Different factors contributed to staff’s ability to attain competency in tracheostomy skills. A significant factor was the use of an apprenticeship model as the primary modality to train staff. While few staff had experienced didactic teaching (Ex. 1), the majority did not participate in a formal or structured training program. Instead, they learned from colleagues by observing, then performing different skills as clinical opportunities arose (Ex. 2). All units had a policy instructing staff to perform tracheostomy care with a colleague for peer support and patient safety (Ex. 2). When there were no TDPP, new staff reviewed unit-based policies and discussed hypothetical patients and situations (Ex. 3). One participant expressed concern that the absence of a structured program can lead to inconsistent training and negative downstream effects on patient care (Ex. 4).

Because the attainment of competency relied heavily on the availability of TDPP, staff had variability in skills due to inconsistent clinical exposure, which was a finding noted in survey responses (Figure 1). Most staff reported limited-to-no clinical experience working with TDPP, including those who worked in their units for many years (Ex. 5). Only a few expressed comfort performing tracheostomy care and stabilizing patients in emergencies because of experiences accrued over many years and/or outpatient work providing home care (Ex. 6).

Staff shared educational strategies to supplement clinical experience, including didactics (Ex. 7), simulations (Ex. 8), and videos (Ex. 9). Just as the use of simulation was endorsed in survey responses, staff advocated for just-in-time learning to review tracheostomy skills with simulation-based education rolling carts in anticipation of a new TDPP (Ex. 10), as well as taking advantage of opportunities for group learning whenever routine tracheostomy care is performed on a patient (Ex. 11). The majority agreed that written materials are least effective for learning over other modalities (Ex. 12). In addition, while simulations are beneficial for skill acquisition and practice in the non-clinical setting, it does not substitute clinical experiences because of conditions that simulations cannot replicate (Ex. 13).

Discussions revealed the prominent role of emotions in learning as a force to drive internal motivation. The anxiety of providing inadequate patient care was the most common emotion expressed, especially by staff who had the least clinical experience working with TDPP (Ex. 14–15). Staff who have witnessed challenging situations emphasized the need to incorporate these types of situations into training (Ex. 16).

### 3.4. Theme: Promoting Caregiver Tracheostomy Skill Development

Staff shared that the degree of caregiver engagement impacted the quality of caregiver education. Staff found joy helping caregivers transition home and enjoyed working with caregivers who showed commitment to achieving this goal (Ex. 17). However, there were times when they faced challenges in motivating caregivers to be involved in their child’s tracheostomy care. One participant shared that it was important to show curiosity to understand challenges so that they know how to best help caregivers (Ex. 18). At times, what staff may perceive as a lack of motivation stemmed from the fear of caring for their child with a tracheostomy. Staff expressed the need to challenge caregivers to step outside of their comfort zone to be involved so that they may develop their skills in incremental steps towards independent performance (Ex. 19).

While a number of methods are utilized to teach caregivers, staff noted inconsistent approaches, revealing the need for the standardization of caregiver education. All caregivers receive a binder of information that they used for education (Ex. 20), but staff expressed the need to better document caregiver attainment of different skills (Ex. 21). Staff helped caregivers practice routine tracheostomy skills on task trainers before performing on their child (Ex. 22). However, staff who provide teaching at the patient’s bedside did not consistently cover more complex scenarios, including emergency situations requiring cardiopulmonary resuscitation (Ex. 22–23). For these types of situations, staff advocated for using simulations to prepare caregivers, especially recognizing that emotions can contribute to the stress of stabilizing a child at home (Ex. 24–25). Most staff shared the anxiety of missing critical information or forgetting to teach high-stakes skills before discharge home (Ex. 26).

With this expressed anxiety, participants discussed staff confidence in teaching caregivers. Staff shared being more comfortable teaching routine tracheostomy skills over emergency situations because of the frequency that they have performed these skills (Ex. 27). Some shared not knowing long-term tracheostomy outcomes or some of the day-to-day considerations of caring for a TDPP at home so that they may provide counseling and answer caregiver questions (Ex. 28). One participant suggested aligning the staff and caregiver education so that staff may have a more standardized approach in their teaching, which would also increase their comfort in teaching (Ex. 29).

### 3.5. Theme: Building Caregiver Preparedness for Home Life

In addition to teaching tracheostomy skills, staff recognized the importance of helping caregivers prepare mentally for home life to help support caregiver confidence and self-efficacy. Staff had to balance caregivers’ development of essential life-saving skills without compromising their confidence that they would be able to perform these skills independently in emergencies (Ex. 30). When possible, staff involved caregivers in such situations so that they can coach them (Ex. 31). However, in the absence of experiencing emergencies in the hospital, one participant suggested involving caregivers in team simulations so that they may learn from staff (Ex. 32).

Staff discussed that identifying community resources was very important to help caregivers prepare and adjust to home life. In the hospital, many caregivers learn quickly that they need to develop managerial skills to identify, hire, and later supervise home nurses (Ex. 33). While staff cannot find and hire nurses for caregivers, staff can help by connecting caregivers to resources such as social media (Ex. 34). Many staff shared that they do not fully understand all the logistics and challenges that families face at home and expressed concern that socioeconomic factors can cause unnecessary hardships (Ex. 35). Some challenges may be mitigated or resolved by connecting caregivers with other families who can offer their lived experience and support (Ex. 34). In some areas, there may be facilities that offer day services for TDPP so that caregivers may retain some daily routine and respite (Ex. 36).

### 3.6. Conceptual Framework

**Figure 2:**
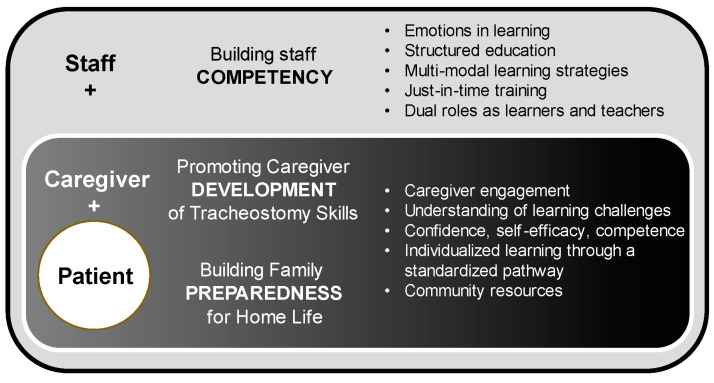
Conceptualization framework. Schematic illustrating the factors (right column) that contribute to providing care to the tracheostomy-dependent pediatric patient in the hospital setting, which include the different needs of staff, caregivers, and their children as patients to effectively attain the goals (themes identified from the focus groups, middle column) and ultimately provide safe patient care and train and support caregivers for the transition home.

The in-depth analysis of staff experiences, which were drawn from survey responses and focus group discussions, led to the development of a framework that conceptualized the factors that contributed to providing care to TDPPs in the hospital (Figure 2). Staff expressed a keen awareness of their responsibilities to themselves, the caregivers, and their patients to provide safe patient care and effectively prepare caregivers for their transition home. They showed vulnerability by sharing anxiety and concern about developing their own competencies and stabilizing patients during emergencies. These emotions magnify with the responsibility of training caregivers, for whom they feared emergencies that caregivers would need to manage as the lone responder for their child at home. These emotions drive staff motivation to identify gaps in knowledge and skills, as well as identify potential educational opportunities and tools to help them become more effective learners and teachers. Staff communicated the need for multi-modal learning strategies that prioritized clinical opportunities and just-in-time training with simulation. They advocated for a similar approach for caregivers and for the need to provide individualized learning based on a standardized pathway. In addition, staff conveyed the importance of understanding caregiver emotions, socioeconomic factors, and community resources that may impact their readiness to learn, attain tracheostomy skills, and build confidence, self-efficacy, and competence.

## 4. Discussion

We performed a mixed-methods study as a part of a needs assessment to understand staff experiences of providing patient care to TDPP. Our study revealed several important findings that impacted staff education and their ability to develop expertise in patient care, as well as their role in training caregivers who ultimately assume responsibility for their child’s care after they transition home from the hospital.

Perhaps the most striking finding was that staff ranged in experience working with TDPP and had limited ongoing clinical opportunities to learn and maintain their competency in providing care to this patient population. Clinical experience and confidence may be limited in part by years of clinical service, with junior staff having less cumulative exposure. This was noted in a survey study by Pritchett et al., in which nurses with <5 years of clinical experience are less likely to express comfort performing tracheostomy skills [21]. While years of clinical experience is an important factor, the frequency of ongoing clinical opportunities also contributes to staff experience. In our study, 59% of staff had not performed a variety of tracheostomy skills or participated in an airway emergency in >1 year (Table 1, Figure 1). This was most likely due to the large number of staff relative to the small number of TDPP. This aligns with Mahfoz’s findings, which suggest that individuals who frequently work with tracheostomies demonstrate greater confidence in their ability to manage and care for them compared to those with less experience [22]. Even though TDPP become chronic patients with extended hospitalizations [36,37], most staff experiences remain sporadic without the ability to maintain a regular or longitudinal experience with them.

With the challenge of limited clinical exposure, building staff competencies in tracheostomy care was a central focus group theme identified in this study (Table 4). Attaining experience solely through an apprenticeship model of learning is not a feasible approach to training and maintaining competency [38,39]. This challenge emphasizes the need to develop a structured program that continues to prioritize learning in the clinical setting while offering regular nonclinical professional development opportunities that support the attainment and retention of knowledge and tracheostomy skills. Because staff have diverse learning needs and different work schedules, a structured program would ideally provide synchronous and asynchronous learning utilizing different educational modalities that include simulation-based training. In our study, staff expressed a strong desire for experiential learning with the use of low-fidelity task trainers for technical skills and more high-fidelity team simulations of complex clinical scenarios for communication and teamwork skills, which have been shown to be effective educational interventions in other types of high-acuity, low-occurrence clinical situations [2,39,40,41].

Educational content should also be informed by frontline staff who work directly with TDPP and caregivers. In a survey study by Hewitt-Taylor, nurses and managers identified general areas of education needed for tracheostomy care, such as communication, feeding, pain management, and resuscitation skills [20]. In our study, staff identified specific tracheostomy skills needed for inpatient care, highlighting skills such as changing tracheostomy tubes, using home ventilators, and responding to alarms on a home ventilator as important (Table 2). In addition, staff shared different types of clinical situations for which they desired more training and practice working as a team. Expressions of anxiety and fear recurred in all focus groups. Even the most experienced staff expressed anxiety about being fully prepared to stabilize TDPP during emergencies, such as airway bleeding, tracheostomy dislodgement, and tracheostomy obstruction. These emotions can serve an important role in individual professional development by activating learning and driving internal motivation to self-identify individual learning needs [42].

In the absence of national or international standards to train healthcare professionals on the care of TDPP, staff ideas for training may be augmented by published studies conducted as single-center educational interventions [12,13,14,24,25,26,27,43]. Studies reporting the use of simulation to train pediatric staff on managing tracheostomy emergencies such as inadvertent decannulation and tracheostomy tube occlusion have increased in recent years [12,13,27,43]. In addition, in a recent study by Schiff et al., a modified Delphi process was utilized to develop an education and assessment tool for a simulated tracheostomy emergency scenario [44]. Apart from the use of simulation, other types of educational interventions also include online modules to allow for asynchronous learning and airway placards as visual educational tools [14,15]. Studies largely focus on improving confidence and knowledge outcomes [12,13,14,15,24,25,27], with some studies also assessing performance skills during a simulation or patient outcomes, such as transfer to an intensive care unit or hospital readmission rates [15,26,27,41]. More studies that evaluate the effectiveness of educational interventions on clinical outcomes are needed.

Tied to the focus group theme of building staff competencies in tracheostomy care is staff awareness that their foundational knowledge and skills are important to promoting caregiver development of tracheostomy skills and building caregiver preparedness for home life, which are also important focus group themes identified in this study (Table 4). Staff during our focus groups were keenly aware of their dual roles as learners and educators. They strived to be effective teachers by developing their own confidence and skills to teach but also expressed anxiety that their own limited clinical experience may lead to gaps in the education they provide caregivers at the bedside. Part of this challenge is the lack of standardized guidelines that may be utilized as a framework to train staff and caregivers who care for TDPP. As a result, hospitals have variation in clinical tracheostomy care protocols and standards, which impacts the type of education provided [45,46]. Developing standardized parallel curricula for staff and caregivers that align the specific learning objectives and competencies that staff need to achieve with those that staff in turn need to train caregivers can help staff envision how to provide that education to caregivers. Such curricula should also incorporate education on home life and available community resources to help staff better understand potential challenges that families face when they bring their child home [17,47].

Education of staff and caregivers may require a different approach in clinical care. Even with a robust training program, many staff who work in large institutions would not be able to attain expertise without many years of consistent clinical experience. Some pediatric units may need to develop a specialized team dedicated to TDPP and caregiver education so that a smaller group of staff have increased opportunities to achieve needed expertise, as well as work together in quality improvement initiatives that would ultimately help standardize practice based on local institutional experience and available national evidence. A partnership with patient safety specialists ensures that educational content aligns with areas of clinical need. Examples of such programs that focus on TDPP have demonstrated decreased adverse events, length of stay, and readmissions [19,41,48,49]. In addition, while there remains limited, high-quality evidence supporting the use of standardized, bundled tracheostomy care, there is an opportunity for different institutions to collaborate to share educational resources, learn from each other, and engage in quality improvement and/or research to evaluate patient outcomes [8,50].

## 5. Limitations

The results of our study reflect staff experiences at a single center. While we reached thematic saturation in analyses of focus group transcripts, healthcare professionals from other institutions may have different experiences working with TDPP, and therefore may have other perceptions of their educational needs. While participants proportionately represented the number of staff who work in different intensive care units of the children’s hospital, we did not include staff who worked in the general pediatric units because the majority of TDPP reside in intensive care units. In addition, the perspectives of caregivers and home care nurses would be important to include for a more comprehensive view of providing care to TDPP. A multicenter study that includes staff who work in inpatient pediatric units with a range of few-to-many TDPPs, as well as home care nurses who work in outpatient settings, would provide a more robust understanding of different educational needs. In addition, a study focused on caregiver experiences, including their training, preparations for the transition home, and their interactions with hospital staff to achieve these goals could strengthen the development of an educational program.

## 6. Conclusions

One of the main findings of our study was that staff who provided care to TDPP had limited ongoing clinical opportunities to learn and maintain their competency in tracheostomy skills. It is not feasible for staff to attain experience and maintain competency solely through an apprenticeship model of learning. There is a need to develop a structured program that continues to prioritize learning in the clinical setting while offering regular non-clinical professional development opportunities that support the attainment and retention of knowledge and tracheostomy skills.

## Figures and Tables

**Table 1:** Characteristics of survey and focus groups participants.

Characteristic	Survey Participants	Focus Group Participants
	n (%)	n (%)
	n = 152	n = 43
**Professional role**		
Advanced practice provider	5 (3)	10 (23)
Physician (attending or fellow)	23 (15)	10 (23)
Nurse	115 (76)	21 (49)
Respiratory therapist	8 (5)	2 (5)
Other	1 (1)	0 (0)
**Location of patient care duties**		
Neonatal intensive care unit	93 (61)	26 (60)
Pediatric cardiac intensive care unit	22 (15)	9 (21)
Pediatric intensive care unit	28 (18)	8 (19)
Multiple locations	9 (6)	0 (0)
**Experience in professional role**		
<2 years	29 (19)	
2–5 years	37 (24)	
6–10 years	35 (23)	
>10 years	52 (34)	
**Experience working with tracheostomy-dependent children**		
None	14 (9)	
<2 years	26 (17)	
2–5 years	31 (21)	
6–10 years	37 (24)	
>10 years	44 (29)	

**Table 2:** Staff confidence performing tracheostomy skills.

Skill	All Staff	Staff Categorized by Experience Working with Trach-Dependent Children					*p* Value
	n = 148						
		0 y	<2 y	2–5 y	6–10 y	>10 y	
		n = 14	n = 26	n = 30	n = 35	n = 43	
Suctioning via tracheostomy	5 (3, 3)	2 (1, 2)	4 (3, 5)	5 (4, 6)	5 (4, 6)	5 (4, 6)	<0.0001
Assessing for skin breakdown around the tracheostomy	5 (3, 6)	2 (2, 3)	4 (3, 5)	5 (4, 6)	5 (4, 6)	5 (4, 5)	<0.0001
Changing tracheostomy ties	4 (2, 6)	2 (1, 2)	3 (2, 5)	5 (3, 6)	5 (3, 6)	5 (3, 5)	<0.0001
Changing the tracheostomy dressing	4 (3, 6)	2 (2, 2)	4 (2, 5)	5 (4, 6)	5 (4, 6)	5 (3, 5)	<0.0001
Responding to an airway emergency (bradycardia, desaturation)	4 (3, 5)	2 (1, 2)	3 (2, 4)	4 (3, 5)	5 (4, 6)	5 (4, 5)	<0.0001
Recognizing and responding to tracheostomy tube dislodgment	4 (2, 5)	1 (1, 2)	2 (2, 4)	4 (2, 5)	5 (3, 6)	4 (4, 5)	<0.0001

Abbreviation: y is years. Data are expressed in medians (Q1, Q3). Different years of experience were analyzed using the Kruskal–Wallis nonparametric test. Staff rated their confidence using a 6-point Likert scale: (1) not at all confident, (2) slightly confident, (3) moderately confident, (4) quite confident, (5) very confident, and (6) extremely confident. Each Likert rating level was defined. Shaded rows: skills that even the most experienced staff reported the least confidence in performing included changing a trach tube, using a home ventilator, and responding to alarms on a home ventilator.

**Table 3:** Staff ratings of the importance for the additional education of specific tracheostomy skills.

Skill	All Staff n = 149	Staff Categorized by Experience Working with Tracheostomy-Dependent Children					*p* Value
		0 y	<2 y	2–5 y	6–10 y	>10 y	
		n = 14	n = 26	n = 31	n = 37	n = 44	
Suctioning via tracheostomy	3 (2, 4)	4 (4, 5)	3 (2, 4)	2 (2, 4)	2 (1, 3)	3 (1, 4)	0.0001
Assessing for skin breakdown around the tracheostomy	3 (2, 4)	4 (3, 5)	3 (2, 4)	2 (1, 4)	2 (1, 3)	3 (2, 4)	0.001
Changing tracheostomy ties	3 (2, 4)	4 (4, 5)	4 (3, 5)	2 (1, 3)	2 (1, 3)	3 (1, 4)	0.0001
Changing the tracheostomy dressing	2 (2, 4)	4 (4, 5)	3 (2, 5)	2 (1, 3)	2 (1, 3)	2 (1, 3)	<0.001
Responding to an airway emergency (bradycardia, desaturation)	4 (3, 5)	5 (4, 5)	5 (3, 5)	4 (3, 5)	4 (2, 5)	4 (2, 4)	0.001
Recognizing and responding to tracheostomy tube dislodgment	4 (3, 5)	4 (4, 5)	5 (3, 5)	4 (4, 5)	4 (2, 5)	4 (3, 50	<0.05
Changing a tracheostomy tube	4 (3, 5)	4 (4, 5)	5 (3, 5)	4 (3, 5)	4 (2, 4)	3 (2, 5)	0.01
Using a home ventilator	4 (3, 4)	4 (3, 5)	4 (3, 5)	4 (3, 5)	3 (2, 4)	4 (3, 5)	<0.05
Responding to alarms on a home ventilator	4 (3, 4)	4 (4, 5)	4 (3, 5)	4 (4, 5)	3 (2, 4)	4 (3, 5)	<0.01
Teaching caregivers tracheostomy care	4 (3, 5)	4 (4, 5)	4 (3, 5)	4 (3, 5)	4 (2, 4)	4 (3, 4)	0.1

Abbreviation: y is years. Data are expressed as medians (Q1, Q3). Different years of experience were analyzed using the Kruskal–Wallis nonparametric test. Staff rated importance using a 5-point Likert scale: (1) not at all important, (2) slightly important, (3) moderately important, (4) quite important, and (5) extremely important. Each Likert rating level was defined. Shaded rows: these are skills that staff with different experience levels rated important for additional education.

**Table 4:** Themes, subthemes and focus group excerpts illustrating staff experiences providing care to trach-dependent pediatric patients and supporting their caregivers.

Themes and Subthemes	Focus Group Excerpts
**Theme: Building Staff Competencies in Trach Care**	
Apprenticeship model	I just did the [orientation] class… It was super educational, and I feel like it went over everything. But there was not anything hands-on, as far as having us actually put our hands on trach ties or an actual trach. We just didn’t have time for it, and I feel like they could be like, “Oh yeah, we went over trach change, trach ties, all that stuff.” But that’s different from actually having to thread your ties… There wasn’t anything formal… You get a baby, and you learn from somebody who knows what they’re doing… They help you out. We’re not supposed to do trach care alone, and we’re not supposed to change ties alone. We always have one or two other staff members helping us out. The respiratory therapists are available to help us out if necessary. Nurses teach the other one-on-one whenever there was a trach, which was very random. Sometimes you’d go months and months and months without a trach. I find that when precepting, sometimes it’s a matter of waves when we have trachs or when we don’t. Sometimes we don’t have any. So, when we’re trying to teach it, we’re just doing it based on policies, and it’s just hypothetical. I think the thing that I worry most about is the consistency in teaching and training and patient care when we don’t have that many trachs… The number of people who get experience discharging a trach kid is relatively small, particularly relative to the size of our team.
Variability in skills due to inconsistent clinical exposure	Even though I’ve been here for a long time, I have very little experience with a baby with a trach. I almost feel like a newer nurse… I’ve never been involved in a trach change, and I’ve never had to place a dislodged trach back in and do that kind of troubleshooting. So, it’s the fear of that happening because I haven’t lived it and gone through it. I personally feel comfortable in an emergency, but I think that there are a lot of people who don’t. I think the fact I’ve done it for home care has helped my comfort zone a lot. I think that everybody else, especially all the newer people who haven’t ever laid eyes on a trach, need to learn emergency stuff right along with all the basic stuff.
Educational strategies: - Didactics - Simulations - Videos - Just-in-time teaching - Written information - Clinical experience	Teaching from the surgical nurse practitioner about the trach sizes, how to put in the trach, [what to do] if it does come out is helpful… I know she’s done a couple of lectures for us. Even the surgical attending has done some stuff for us… It has been a while. I feel like when you do it only once in a while, it’s always harder, but having some kind of mannequin in teaching can actually boost that confidence level in everybody. I found that sometimes short videos are helpful initially to look at the whole technique first, then try it on a mannequin and do it on the baby, as a progression. We usually have forewarning when a trach is going to be happening, so perhaps we need to seize the days leading up to a trach being inserted to pull as many people together and do just-in-time [education with] rolling carts. We can do it in the neighborhood that the baby is in and say, “You all know this baby’s going to be trached in three days. Let’s all get on the same page and remind ourselves of what our care is. The next time we have a trach patient, it would be cool if somebody sent out a text like, “Anybody want to see trach care?” or “We’re changing this cannula,” because it’s real life, and it’s not like you have to imagine going through a policy. You’re actually seeing it done on a patient. You’re seeing possibly things go wrong as well, but you have support from the bedside nurse, respiratory therapy, and maybe even the parents. So, it would be a bedside teaching session on an actual patient, with parent permission. Written material is probably the least helpful in whatever form. Most helpful is probably hands-on experience. A video is probably somewhere in between because it allows visualization. Websites can be very unhelpful, because there’s a lot of mixed information, which sometimes can be more damaging than helpful. I feel like for trach changes, it would be more beneficial on a real baby. I mean you can get the gist on a mannequin, but it feels quite different. You’re not going to get the sweaty screaming baby that is moving all over the place… It’s just not the same. You don’t get the fat rolls and the skin moving.
Role of emotions in learning	[Changing a trach] is like riding a bike until it doesn’t go back in, right? I would be nervous if somebody came in. I would love to change one, because I haven’t changed one in years honestly… I think even just watching would be helpful. In talking to the newer nurses, they’ve all said they would freak out if a trach came out and wouldn’t know how to handle it. I know that was one of my big fears, probably the first or so times I took care of a kid with a trach. For a couple of post-op patients with fresh trachs, the trach became dislodged. There was excessive bleeding everywhere. They couldn’t get the trach in initially because they couldn’t visualize. We called ENT. We ended up plugging the hole where the trach was and intubating through the mouth until ENT came down… Those are the most concerning. They bleed so much you don’t get to see the airway, and it’s pretty emergent and scary. I think education around that type of [situation] would be very important.
**Theme: Promoting Caregiver Development of Trach Skills**	
Caregiver engagement	What’s my favorite part about [teaching]? When [caregivers] do a good job—when they feel proud of themselves and are ready to take their kid home. That’s the ultimate goal. You want them to be confident. The more they do it, the more confident that they are. With one baby, we had the mom do [a trach change] one week and the dad do it the next week—taking turns. The better that they work together, the better the whole thing goes. I think it’s difficult when you get a sense from the parents that they don’t care. They’re not concentrating or focusing on what you’re saying… I think a lot of it actually comes from being fearful, and I think digging down and talking about what is giving them anxiety about going home with a trach baby will help us get a lot further. I think sometimes they’re reluctant just because they’re honestly scared. I think we have to push them to be a lot more hands on. I think there are a lot of times that we take that responsibility from the family. Then we do a lot of the care, and we don’t push them to make sure that they’re here every day and doing this. I think if they did that more consistently, they would be more comfortable going home. Nursing’s not going to go home with them and do all this for them. So, I think pushing them to really be involved, even if it’s uncomfortable, would be helpful in the long run.
Standardization of caregiver education - Written information - Documentation - Simulations - Emergency situations	When I’m trying to reinforce education and I know that the parents have their trach binder, I often will be like, “What do you have?… Do you have a picture of what you’re supposed to be doing?… Let’s flip to it, and I can clarify what this means. We need to be better at documenting… It’s like, “Oh, have these parents given these meds or not, because I can’t tell,” and then some people say, “They know what they’re doing,” but, literally no one has charted that. I think the weekly trach changes sets [caregivers] up with a good sequence of what to do. We talk about doing demos with parents on dolls… I don’t do a lot of [scenarios] with the families. We just do the basic trach changes. We don’t really teach them emergency situations. What if you went to check on the baby, and the baby’s trach is out? What do you do first? Do you call 911? Do you try to put the trach in? Do you do CPR? I’ve never discussed that with a family before. I go through emergency scenarios with them. “If this happens, this is what we do.” We teach them CPR. I teach them that if you’re doing CPR, and you can’t get a new trach in, you try your one size smaller. Then we teach them how to bag with covering up the stoma. They get all of that before going home. Simulation alone obviously wouldn’t be sufficient, but it does seem like a great way to amplify the family’s learning, reinforce it. I think simulation would be great, because I think a lot of times, the routine trach changes go really smoothly. But in the setting of their own house, that obviously is very stressful and anxiety-provoking and might put a barrier to their performance. I don’t know if there’s any way to simulate it where it ends up being a little bit more stressful so that they kind of know what it will be like at home, with their adrenaline pumping and everything. It may seem or appear to us that they [caregivers] are competent in their capabilities of caring for their child with a trach. Unfortunately, we sometimes hear of kids who pass away at home if their trach dislodged or something. It might not have been the parents’ fault, but I just worry that we missed something that we could have taught them. Or they didn’t feel comfortable when they left and just didn’t say anything to us.
Staff confidence to teach caregivers	With cleaning and suctioning, the reason we feel okay teaching it is because we’re so personally comfortable with doing it. If we became more personally comfortable with doing trach changes and emergency-type things, then we’ll feel more comfortable teaching it. When I’m discharging a cardiac kid or a kid with a gastrostomy tube, I feel comfortable enough where I can reassure [caregivers]… and chat with them about what to expect when they go home. What is life going to be like upon discharge? What precautions do they have to do? What activities aren’t they going to be able to do for a while? But for a kid with a trach, I don’t really know. I can imagine if I were a parent going home with a kid with a trach, I think I would feel more anxious if I’d asked a nurse, “Hey, what is going to be my experience when I go home?” and I’m not able to really tell them much of anything. That would probably make my anxiety as a parent even worse. I wonder if our education that we received biannually was literally the education that is provided to parents. Same spiel, same scenario, etc… and that would be super useful for us to then not only feel more comfortable ourselves in doing it, but also in teaching it.
**Theme: Building Caregiver Preparedness for Home Life**	
Caregiver confidence and self-efficacy	One of most important things for [caregivers] to understand is the significance of maintaining an airway. You walk that fine line of stressing to them that this is essential and that the airway needs to be maintained for their child to live, but you also want them to feel competent enough in the skills that they’re learning to be able to go home. I’ve had a trach dislodged, and the parent assisted me in getting it back in. I had them deflate the balloon while I replaced it. Then it went in easily and thankfully was easily fixed. They needed my help but was able to take direction and understood when I said to deflate the balloon. They knew how to do that. I do wonder about the utility of incorporating families into our simulations. If we have a family that we know is going to take a trach and vented patient home, it would be nice to have the family incorporated into simulations so they could practice and see what we would do as a skilled health care team. It might give them confidence to know, “Oh, I remember what so-and-so did. Here’s what we should do,” Then they should be able to practice as well.
Community resources	Just think about yourself with the kid with a trach. You become an employer. You have to hire your own nurses—find them, recruit them, interview them—and train them in many cases, because many of the home nurses out there do not have trach experience. So then, this makes it even more important for us in the hospital to train the families properly, because once they go home, they’re the ones who have to do quality control… A huge issue for a lot of these families is getting their [home] nurses. It keeps a lot of kids here [in the hospital] longer. I always tell the families about the Medicaid family Facebook page… so that they can look for nurses, and nurses can look for families. Sometimes families will reach out to each other with common issues, like supplies. It’s taken a lot to understand their limitations—both financially, socioeconomically—and what their resources are when trying to take a child home. Realistically, they’re not the only person that’s taking care of the kid. They might be on a rotating system of not one or two but multiple different people. Financially, how are they really affording the care for this kid? What is their medical literacy level, and how much do they truly understand? There are daycares that would take a kid, like a respite system when [caregivers] are struggling… I think if that doesn’t happen, then very often kids end up in the emergency room for minor complaints, because [their caregivers] are just really exhausted and overwhelmed managing these kids. It’s like running a hospital at home, and I feel parents can be pretty overwhelmed and exhausted by the whole process.

Abbreviations: Trach is tracheostomy.

## Data Availability

The original contributions presented in this study are included in the article/Supplementary Materials. Further inquiries can be directed to the corresponding author.

## Supplementary Materials

The following supporting information can be downloaded at: https://www.mdpi.com/article/10.3390/children12050552/s1. Supplementary Materials S1: Survey questions. Supplementary Materials S2: Focus Group Guide.

## Abbreviations

The following abbreviations are used in this manuscript:

EX	Excerpt
TDPP	Tracheostomy-dependent pediatric patient
