# Supplementary material for: Experiences and Educational Needs of Hospital Staff Providing Care to Tracheostomy-Dependent Pediatric Patients
Source: Children (Basel). 2025 Apr 25;12(5):552. doi: 10.3390/children12050552 (PMC12110650; doi:10.3390/children12050552)
Supplement: Supplementary file 1 [file children-12-00552-s001.zip › children-3540980-supplementary.pdf]

Supplementary Materials S1. Survey questions

You are being asked to complete this survey as a part of an educational needs assessment for care of the tracheostomy-dependent child. This anonymous survey will be used to determine the educational needs of staff caring for this patient population.

Section S1: General Information

1. **Which hospital unit do you primarily work?**
   ___ Neonatal ICU
   ___ Pediatric ICU
   ___ Pediatric Cardiac ICU
   ___ I work in the following multiple units equally:

2. **What is your job title?**
   ___ Registered nurse
   ___ Nurse practitioner, physician assistant
   ___ Fellow, attending
   ___ Discharge coordinator, care coordinator
   ___ Advanced practice registered nurse, clinical nurse leader, nurse educator
   ___ Respiratory therapist
   ___ Other:___________________

3. **How many years of experience do you have as a medical professional (RT, RN, NP, PA, MD, DO, etc.)?**
   ___ < 2 years
   ___ 2-5 years
   ___ 5-10 years
   ___ > 10 years

4. **How many years of experience do you have working with children with technology dependence (trach, g-tube, broviac, home ventilator)?** [multiple choice question]
   ___ None
   ___ < 2 years
   ___ 2-5 years
   ___ 5-10 years
   ___ >10 years

Section S2: Tracheostomies

5. **During the last year, how many patients with tracheostomies have you worked with in your primary hospital unit?**
   ___ None
   ___ 1-3
   ___ > 3

6. **For the following question, *confidence* is defined as the feeling or belief that you can perform the skill well.**

   Please use the following Likert scale:
   1. *Not at all confident* - I would like to watch a colleague perform the skill first
   2. *Slightly confident* - I feel comfortable with a colleague assisting me during the whole skill
   3. *Moderately confident* - I can perform the skill with a colleague assisting me during 50% of the skill
   4. *Quite confident* - I can perform the skill independently, but I might need to ask for help or refer to training materials, checklist, resource nurse, etc.
   5. *Very confident* - I feel comfortable performing the skill independently, but do not feel comfortable teaching the skill to others (colleagues or caregivers)
   6. *Extremely confident* - I feel comfortable performing the skill independently, and can teach the skill to others (colleagues or caregivers)

   Rate your level of confidence for the following tracheostomy and ventilator skills below

   ___ Suctioning via the tracheostomy (knowing how deep, using Ballard suction and/or open suction)
   ___ Assessing for skin breakdown around the tracheostomy
   ___ Changing tracheostomy ties
   ___ Changing the tracheostomy dressing
   ___ Changing the tracheostomy tube
   ___ Recognize and respond to trach dislodgment
   ___ Responding to an airway emergency (bradycardia, desaturation)
   ___ Using a home ventilator
   ___ Responding to alarms on a home ventilator

7. **When was the last time you performed the following skills**?

   Please use the following key:
   1. Within 6 months
   2. 6-12 months
   3. It has been >12 months
   4. I have never performed independently

   ___ Suctioned via the tracheostomy
   ___ Assessed for skin breakdown around the tracheostomy
   ___ Changed tracheostomy ties
   ___ Changed the tracheostomy dressing
   ___ Changed the tracheostomy tube
   ___ Responded to an airway emergency
   ___ Used a home ventilator

8. **Specify how important it is for you to receive additional education for the following skills**:

   Please use the following Likert scale:
   1. Not at all important
   2. Slightly important
   3. Moderately important
   4. Quite important
   5. Extremely important

   ___ Suctioning via the tracheostomy
   ___ Assessing for skin breakdown around the tracheostomy
   ___ Changing tracheostomy ties
   ___ Changing the tracheostomy dressing
   ___ Changing the tracheostomy tube
   ___ Responding to an airway emergency
   ___ Using a Responding to alarms on a home ventilator home ventilator
   ___ Recognizing and responding to a tracheostomy dislodgement
   ___ Teaching caregivers tracheostomy care

Section S3: Wrap-Up

9. **What concerns do you have or what changes would you like to make for the discharge process for technology dependent children?**

   ____

   ____

10. **What other topics you would like education on related to tracheostomy-dependent children?**

    ____

    ____

11. **What any additional education you believe families need such as specific scenarios that should be simulated with them prior to discharge?**

    ____

    ____

12. **Are there any websites or resources that you find helpful when caring for technology dependent children?**

    ____

    ____

13. **For didactic information, how would you like to receive this information? Please list in order of preference.**
    ___ Unit based in-services

___ Classroom lecture/hands on
___ Computer based self-learning modules

Supplementary Materials S2. Focus Group Guide

Introduction:

Thank you for your time and participation in this focus group. The purpose of this focus group is to understand staff experience working with tracheostomy-dependent children and caregivers as they prepare for discharge home from the hospital.

Self-identified training needs:

1. What educational opportunities do you feel would be most helpful related to caring for infants/children with tracheostomies?
2. What educational opportunities do you feel would be least helpful related to caring for infants/children with tracheostomies?
3. What simulations/scenarios would be helpful to practice with a mannequin? Why? What would be the least helpful? Why?
4. What worries you the most about caring for infants/children with tracheostomies?
5. What tracheostomy emergencies have you experienced?
6. How did you handle the emergency situation?
7. Have you been involved in an emergency that you feel could have been handled differently? If so, how?
8. How has past training that you have received prepared you for emergency situations with infants/children with tracheostomies?

Working with caregivers of tracheostomy-dependent infants:

9. How much experience do you have teaching and training caregivers of infants/children with tracheostomies? What has been your experience?
10. What components do you find the most challenging?
11. What components do you enjoy teaching the most?
12. What would help make you feel more confident with teaching/reinforcing concepts for caregivers of infants/children with tracheostomies?
13. What worries you the most about caregivers taking infants/children with tracheostomies home?
14. What do you think are the most important components caregivers should take away from teaching in the hospital?
15. What do you wish you knew more about related to infants/children with tracheostomies being discharged from the hospital?
16. Imagining yourself as a caregiver of infants/children with tracheostomies, what training would you want prior to going home from the hospital? What would you be worried about going home?
17. What family resources would you like to learn more about?
